# Supplementary material for: Ureaplasma Species Modulate Cytokine and Chemokine Responses in Human Brain Microvascular Endothelial Cells
Source: Int J Mol Sci. 2019 Jul 22;20(14):3583. doi: 10.3390/ijms20143583 (PMC6678482; doi:10.3390/ijms20143583)
Supplement: Supplementary file 1 [file ijms-20-03583-s001.pdf]

Supplementary file 1. Readout methods applied to individual mediators.

	**qRT-PCR**	**RNA-sequencing**	**immunoassay**	**flow cytometry**
**CXCL5**	yes	yes	yes	no
**CXCR4**	yes	yes	no	no
**IL-1α**	yes	yes	yes	no
**IL-1β**	yes	yes	yes	no
**IL-1RA**	no	yes	yes	no
**IL-6**	no	yes	yes	no
**IL-8**	yes	yes	yes	yes
**IL-10**	yes	yes	yes	yes
**MCP-1**	yes	yes	yes	no
**MCP-3**	yes	yes	yes	no
**MMP-9**	yes	yes	yes	yes
**TNF-α**	yes	no	yes	no
